# The Mosquito Melanization Response Is Implicated in Defense against the Entomopathogenic Fungus *Beauveria bassiana*

**DOI:** 10.1371/journal.ppat.1003029

**Published:** 2012-11-15

**Authors:** Hassan Yassine, Layla Kamareddine, Mike A. Osta

**Affiliations:** Department of Biology, American University of Beirut, Beirut, Lebanon; Stanford University, United States of America

## Abstract

Mosquito immunity studies have focused mainly on characterizing immune effector mechanisms elicited against parasites, bacteria and more recently, viruses. However, those elicited against entomopathogenic fungi remain poorly understood, despite the ubiquitous nature of these microorganisms and their unique invasion route that bypasses the midgut epithelium, an important immune tissue and physical barrier. Here, we used the malaria vector *Anopheles gambiae* as a model to investigate the role of melanization, a potent immune effector mechanism of arthropods, in mosquito defense against the entomopathogenic fungus *Beauveria bassiana*, using *in vivo* functional genetic analysis and confocal microscopy. The temporal monitoring of fungal growth in mosquitoes injected with *B. bassiana* conidia showed that melanin eventually formed on all stages, including conidia, germ tubes and hyphae, except the single cell hyphal bodies. Nevertheless, melanin rarely aborted the growth of any of these stages and the mycelium continued growing despite being melanized. Silencing *TEP1* and *CLIPA8*, key positive regulators of *Plasmodium* and bacterial melanization in *A. gambiae*, abolished completely melanin formation on hyphae but not on germinating conidia or germ tubes. The detection of a layer of hemocytes surrounding germinating conidia but not hyphae suggested that melanization of early fungal stages is cell-mediated while that of late stages is a humoral response dependent on TEP1 and CLIPA8. Microscopic analysis revealed specific association of TEP1 with surfaces of hyphae and the requirement of both, TEP1 and CLIPA8, for recruiting phenoloxidase to these surfaces. Finally, fungal proliferation was more rapid in *TEP1* and *CLIPA8* knockdown mosquitoes which exhibited increased sensitivity to natural *B. bassiana* infections than controls. In sum, the mosquito melanization response retards significantly *B. bassiana* growth and dissemination, a finding that may be exploited to design transgenic fungi with more potent bio-control activities against mosquitoes.

## Author Summary

Melanization is an important immune response and wound healing mechanism in arthropods that leads to melanin formation and deposition on microbial and wound surfaces, respectively. In the *Anopheles gambiae* mosquito that transmits the malaria parasite *Plasmodium*, melanization is dispensable for parasite killing. Further, we have shown that in *Anopheles gambiae* this immune process does not seem to play a role in defense against bacterial infections, which questions the role of melanization in mosquito immunity and the microbial pressure that drove its evolutionary path. Here, we infected mosquitoes with the entomopathogenic fungus *Beauveria bassiana* to study the role of melanization in anti-fungal defense. We show that mosquito blood cells, as well as specific immune proteins present in the mosquito blood participate in the melanization response to *Beauveria bassiana*. Our results also reveal that melanization does not abort the growth of the fungus but rather retards significantly its proliferation. The hyphal body stages, which are freely circulating single cells that can disseminate the infection appear earlier in mosquitoes exhibiting a compromised melanization response than in control mosquitoes. These findings provide novel insights into *Beauveria bassiana*-*Anopheles gambiae* interactions, which may be exploited to design transgenic fungi with enhanced bio-control potential against mosquitoes.

## Introduction

Melanization is an immediate immune response in arthropods leading to the physical encapsulation of pathogens in a dense melanin coat, and to the generation of toxic metabolites that can harm certain pathogens. It is also a prominent wound healing process manifested by the blackening of the wound area in arthropods. Melanization is triggered by pattern recognition receptors (PRRs) that upon binding pathogen associated molecular patterns (PAMPs) activate a cascade of serine proteases culminating in the proteolytic cleavage and conversion of the prophenoloxidase (PPO) zymogen into active phenoloxidase (PO), the rate limiting enzyme in melanogenesis [1]. The protease cascade acting upstream of PPO involves often a modular protease and several downstream clip-domain serine proteases (CLIPs) [2], [3]. This cascade is under tight temporal regulation by serine protease inhibitors (SRPNs). In the dipterans *Drosophila* and *Anopheles gambiae*, the absence of *SPN43Ac*, also called *necrotic*, [4] and SPRN2 [5], respectively, resulted in the appearance of spontaneous melanotic pseudotumors in adult tissues and a reduced life span, suggesting that aberrant control of melanization imposes a fitness cost on the host. Further, this process is regulated spatially which ensures that melanin formation occurs exclusively on microbial surfaces minimizing collateral damage to the host.

Biochemical studies in *Manduca sexta* [6] and *Tenebrio molitor* [7] revealed that PPO activation is further controlled by the requirement of non-catalytic CLIPs [also known as serine protease homologs (SPHs)] as co-factors for prophenoloxidase activating enzymes (PPAE) to trigger proper processing of PPO into PO. SPHs have substitutions at one or more of the critical His/Asp/Ser triad that renders them non-catalytic. Functional genetic studies in the malaria vector *A. gambiae* revealed that a clip domain-containing SPH termed CLIPA8 is required for the melanization of *Plasmodium berghei* ookinetes in the mosquito midgut [8] and bacteria in the hemocoel [9]. While there is no evidence yet for the direct involvement of CLIPA8 in the processing of PPO, these studies provided a strong genetic evidence for the central role of SPHs in the melanization response *in vivo*.

Several reports have linked melanization to insect defense. In the crustacean *Pacifastacus leniusculus*, PO activity is required for defense against the bacterial pathogen *Aeromonas hydrophila*: RNAi-mediated silencing of PO was associated with increased susceptibility to *A. hydrophila* while silencing pacifastin, an inhibitor of the crayfish PO cascade, resulted in increased resistance to the bacterium [10]. The fact that *Photorhabdus* bacteria pathogenic to *M. sexta* [11], and polydnaviruses carried by female parasitoid wasps [12], evolved independent specific strategies to counteract the host melanization response is a further indication of the importance of this response in insect defense.

Previous genetic studies in the model dipteran *Drosophila* revealed that the melanization response does not seem to be critical for survival of flies after bacterial or fungal infections [13], [14]; rather, melanization seems to enhance the effectiveness of subsequent immune reactions in the fly by weakening a microbial infection at an early stage [14]. However, a more recent study, employing a larger panel of bacterial species, demonstrated an important role for this immune process in modulating tolerance as well as resistance of the fly to specific bacterial infections [15]. Abolishing PO activity in the malaria vector *A. gambiae*, by silencing CLIPA8, did not affect mosquito survival after infections with *Escherichia coli* or *Staphylococcus aureus* [9]. Both bacterial species were cleared from CLIPA8-silenced mosquitoes as efficiently as from controls suggesting that melanization is not critical for anti-bacterial defense in the mosquito. In *A. gambiae*, the melanization response to *P. berghei* is also controlled by CLIPA8 [8], in addition to the complement-like protein TEP1 [16] and two leucine-rich immune proteins, LRIM1 [17] and APL1C [18], [19]. The latter two proteins form an obligate disulfide-linked heterodimer in the mosquito hemolymph that interacts with and stabilizes a cleaved form of TEP1 [20], [21]. In addition to triggering ookinete lysis in the basal labyrinth of the midgut epithelium [16]–[18], the TEP1/LRIM1/APL1C complex (henceforth TEP1 complex) is also required for the melanotic response to ookinetes in refractory mosquito genotypes [16], [17] as well as to bacteria injected directly into the hemolymph (unpublished data). Nevertheless, wildtype laboratory and field caught *A. gambiae* mosquitoes rarely melanize malaria parasites [22] and melanization does not seem to be important for *A. gambiae* anti-bacterial defense [9], questioning the role of this response in mosquito immunity.

Research on mosquito immunity has focused mainly on parasites, bacteria (reviewed in [23]) and lately viruses [24]–[26], whereas entomopathogenic fungi received little attention despite their ubiquitous nature and their route of infection which unlike other microbial classes does not require ingestion by the host. Rather, these fungi infect by direct penetration through the mosquito cuticle into the hemolymph. This mode of infection is particularly attractive for immunity studies because it does not require artificial injection of the microbe into the hemolymph. It also bypasses the midgut epithelium which was shown recently to engage in promoting complement-mediated ookinete lysis in the basal labyrinth of the midgut epithelium, by triggering intracellular nitration of ookinete surface proteins [27]. Here, we investigate the role of melanization in defense against natural infections with the entomopathogenic fungus *B. bassiana* and provide novel insights into the cellular and molecular mechanisms triggering fungal melanization *in vivo*.

## Results

### *Beauveria bassiana* infection triggers the melanization response of *Anopheles gambiae*

**Figure 1:**
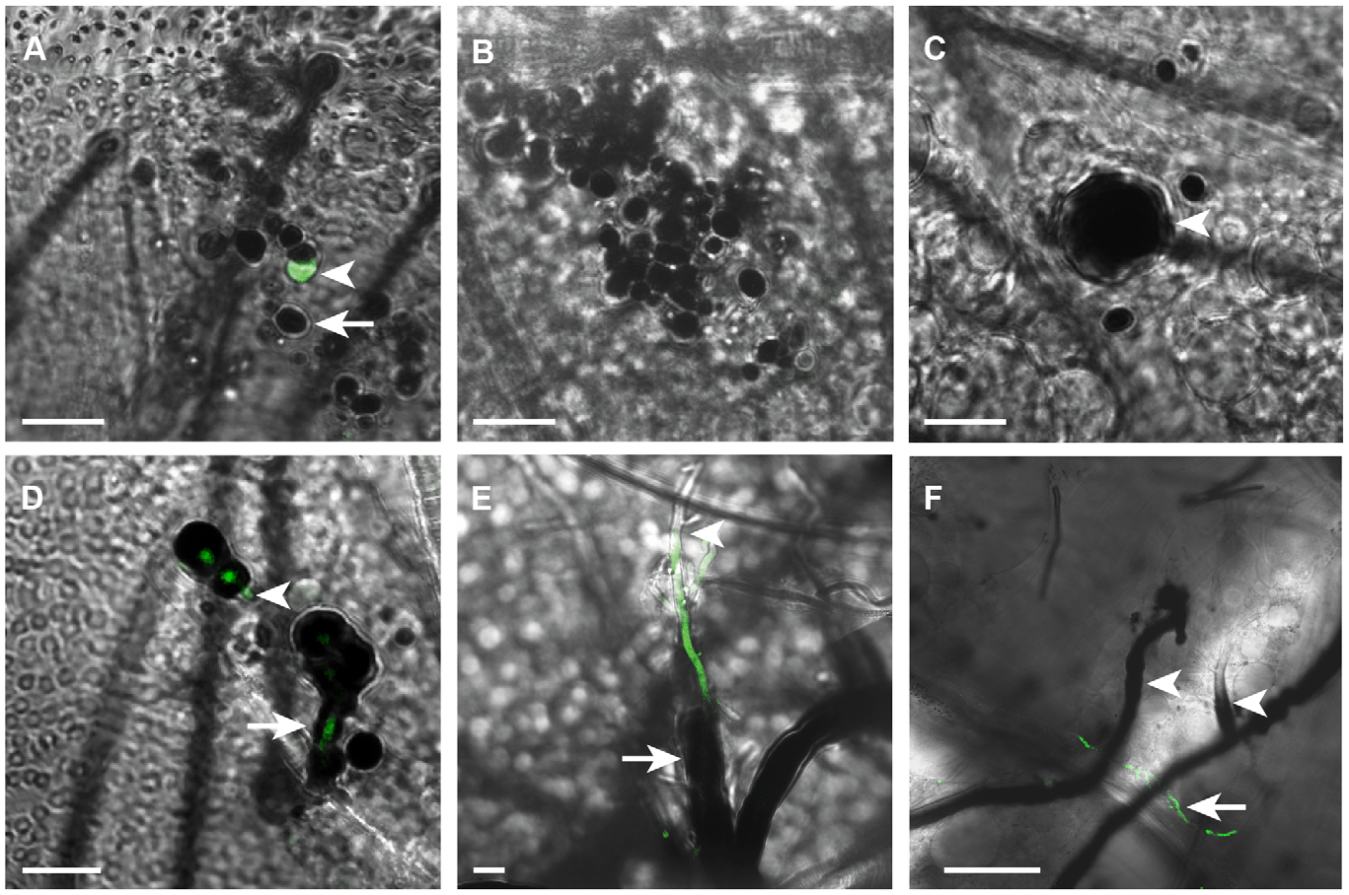
Mosquito melanization response to *B. bassiana* developmental stages. Merged fluorescent and bright field images of abdomens dissected from mosquitoes at (A) 1 hr, (B) 6 h, (C) 12 h, (D) 24 h and (E and F) 48 h following the injection of each with 200 conidia of GFP-expressing *B. bassiana*. (A) Conidia were rapidly melanized (arrow) 1 h post-injection (pi); few non-melanized conidia were detected at that time point (arrowhead). (B) All conidia were melanized at 6 h pi. (C) An enlarged conidium germinating within the melanotic capsule (arrowhead) at 12 h pi. (D) A germ tube breaking through the melanin coat at 24 h pi (arrowhead) and another elongating with concomitant melanin formation around its wall (arrow). (E) Partially melanized hypha in a mycelium at 48 h pi showing absence of melanin at the apical part (arrowhead) and presence of a thick melanin coat around the basal part (arrow) (F) A low magnification image showing extensive melanization of hyphae (arrowheads) in the growing mycelium at 48 h pi, with few GFP-expressing, non-melanized hyphae detected (arrow). h, hour; GFP-expressing *B. bassiana* (Green). Scale bars are 10 µm in A–E and 50 µm in F.

Mosquito immune responses to entomopathogenic fungi such as *B. bassiana* remain poorly understood. We carried a meticulous microscopic analysis of *B. bassiana* development in adult *A. gambiae* mosquitoes to determine whether melanization is also triggered in this model infection system and against which stages. To facilitate detection of the fungus in dissected mosquito abdomens we utilized a GFP-expressing strain of *B. bassiana* [28]. Individual mosquitoes were injected intrathoracically with 200 freshly prepared conidia (spores) and fungal development was monitored in dissected abdomens at 1, 6, 12, 24 and 48 h post-infection (pi). In these assays, mosquitoes were infected by injecting conidia rather than by the natural route (tarsal contact with spores), because in the former mycelial growth was frequently observed in dissected abdomens which was the not case in natural infections. Most conidia were rapidly melanized at 1 hr pi; these appeared black since GFP was masked by the melanotic capsule (Figure 1A). Only rare non-melanized conidia were observed at that time point. At 6 h pi all conidia were melanized (Figure 1B), however, at later time points, some melanized conidia exhibited an enlarged size indicating that germination was taking place within the melanotic capsules (Figure 1C). Indeed, at 24 h pi, germ tubes started emerging from melanized conidia concomitant with melanin formation around their walls (Figure 1D). Two days pi, melanin was also detected on several hyphae that emerged from germ tubes, while few hyphae remained non-melanized (Figures 1E and 1F). Altogether, our data revealed that conidia, germ tubes and hyphae were efficiently melanized in *A. gambiae* mosquitoes, yet this immune reaction was not sufficient to abort completely the development of the mycelium. However, we noticed that hyphal bodies, yeast-like single-cells that differentiate from growing hyphae, were rare and sometimes absent in abdomens at 48 h post conidia injection, whereas these stages were commonly present in the hemolymph of other insect species at the same time point [29], [30]. This suggests that melanization might be retarding the growth of the fungus in the mosquito.

### Cellular and humoral melanotic responses elicited against *B. bassiana* in adult mosquitoes

**Figure 2:**
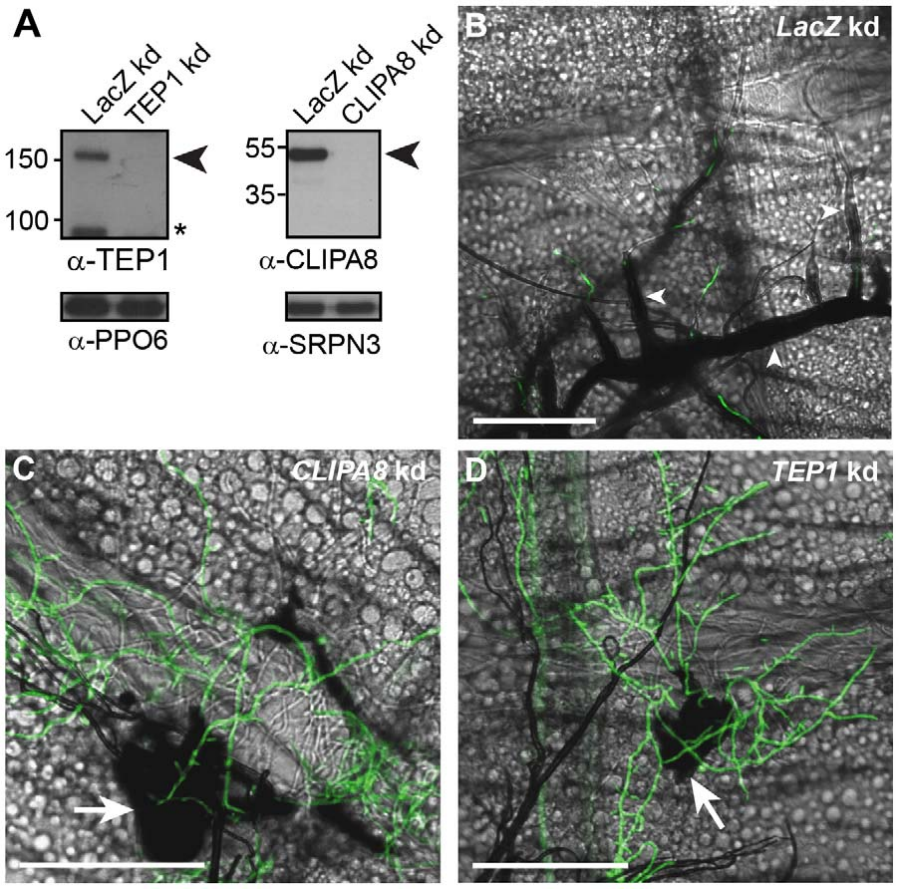
TEP1 and CLIPA8 are required for the melanization of hyphae. (A) Western blot analysis showing CLIPA8 and TEP1 depletion in the hemolymph four days after silencing their corresponding genes by RNAi. *Left*, arrowhead indicates full-length TEP1 (TEP1-F) and asterisk denote the cleaved form of TEP1 (TEP1-C); *Right*, arrowhead indicates full length CLIPA8. PPO6 and SRPN3 were used as loading controls. (B) Abdomens dissected from *LacZ* kd mosquitoes at 48 h post-injection of 200 conidia of GFP-expressing *B. bassiana* (Green), show intense melanization of hyphae (arrowheads) (C and D) Absence of melanin around hyphae in *CLIPA8* and *TEP1* kd mosquitoes, respectively. Note, however, the strong melanization associated with the base of the mycelium in each of these genotypes (arrows). Scale bars are 100 µm.

The melanization of *P. berghei* in certain refractory *A. gambiae* mosquito genotypes is a humoral response dependent on CLIPA8 [8], and TEP1 complex [16], [17], [20], [21]. In this model system, the midgut basal lamina constitutes a physical barrier that inhibits direct contact between hemocytes and ookinetes residing in the basal labyrinth. Bacteria injected into the hemolymph also elicit a humoral melanotic response dependent on CLIPA8 [9] and TEP1 complex (unpublished data), suggesting that these are core proteins in the mosquito melanization response. To address whether they exhibit similar roles in infections with *B. bassiana*, *TEP1* and *CLIPA8* were silenced in adult female mosquitoes by RNAi knockdown (kd) [31], then individual mosquitoes were injected with 200 conidia of GFP-expressing *B. bassiana*. Mosquito abdomens were dissected two days after spore injection in order to score fungal melanization. Western blot analysis of hemolymph extracts confirmed that *TEP1* and *CLIPA8* were efficiently silenced four days after injection of their corresponding double-stranded RNAs (Figure 2A). In *LacZ* kd control mosquitoes, a thick melanin coat covered the majority of the growing mycelium as expected (Figure 2B). In contrast, hyphal melanization was completely abolished in *CLIPA8* and *TEP1* kd mosquitoes, suggesting that these proteins are indeed core regulators of the melanization response (Figures 2C and 2D, respectively). Intriguingly, in these two genotypes, only the base of the growing mycelium from which hyphae emerged was still melanized as efficiently as in *LacZ* kd controls. These findings were unexpected since the same gene knockdowns completely abolished the melanotic response to *P. berghei* [8], [16] and bacterial infections ([9] and unpublished data).

**Figure 3:**
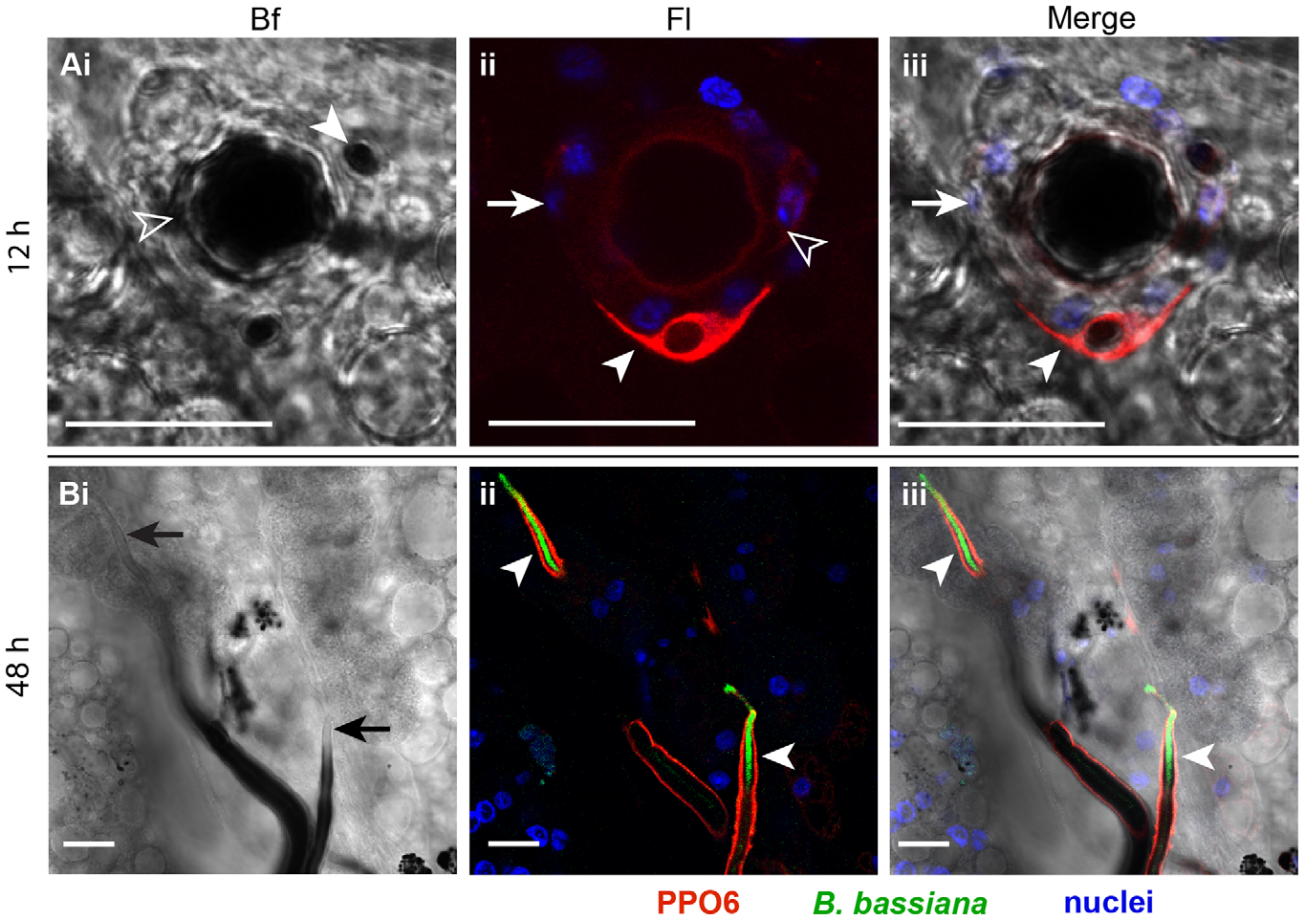
*B. bassiana* infection triggered cellular and humoral melanotic responses in the mosquito. Abdomens were dissected at (A) 12 and (B) 48 h post-injection of each mosquito with 200 conidia of GFP-expressing *B. bassiana* and immunostained with α-PPO6 antibody. Shown are bright field (Bf), fluorescence (Fl), and merged bright field and fluorescence (Merge) images of confocal sections. (A) PPO staining of a germinating conidium. A conidium germinating despite being melanized (Ai, open arrowhead) exhibited an enlarged size compared to a nearby non-germinating spore (Ai, filled arrowhead). A cellular layer of hemocytes surrounding the germinating conidium (Aii and Aiii, arrows). These hemocytes exhibited no or faint PPO signal (Aii, open arrowhead). A hemocyte expressing PPO (Aii and Aiii, filled arrowhead) was resting on top of the cells that surrounded the germinating conidium. (B) PPO staining of hyphae. The apical parts of hyphal filaments exhibited a thin melanin coat (Bi, arrows) but strong PPO signal (Bii and Biii, arrowheads). The absence of hemocytes around these hyphae inform a humoral melanotic response. PPO6 (red), GFP-expressing *B. bassiana* (green), nuclei (blue). All scale bars are 20 µm.

We hypothesized that two distinct mechanisms are driving the melanotic response to the fungus. The first is hemocyte-mediated and targets the early stages of fungal development in the mosquito, including the germinating spores and germ tubes. The second is humoral, dependent on TEP1 and CLIPA8 functions, and targets the hyphae that develop later. To address this point, abdomens dissected from wildtype mosquitoes, at 12 and 48 h after conidia injection were immunostained with polyclonal antibody against PPO6, which is known to be expressed in hemocytes [32], [33]. Abdomens dissected at the earlier time point clearly showed a circular arrangement of hemocytes around enlarged conidia that were apparently germinating within the melanotic capsule (Figure 3A). Most of these hemocytes showed absence of, or a faint PPO signal possibly because they have exhausted their PPO content in the struggle against the germinating conidium. Alternatively, some of these hemocyte may not express PPO. A hemocyte strongly expressing PPO was resting on top of two other hemocytes that are in direct contact with the conidium (Figures 3A), suggesting that hemocytes recruited to the germinating spore may form more than one layer around it attempting to abort its growth, pretty much similar to nodule formation in larger insects. At 48 h after infection, no hemocytes were detected in close proximity to melanized hyphae supporting the humoral nature of this response (Figure 3B). The hyphal tips from where growth occurs exhibited a thin, often barely detectable layer of melanin, but a strong PPO signal (Figure 3B), suggesting that melanin biosynthetic reactions were still particularly active at these foci. Yet, the whole was taking place in the absence of hemocytes from the hyphal tips.

**Figure 4:**
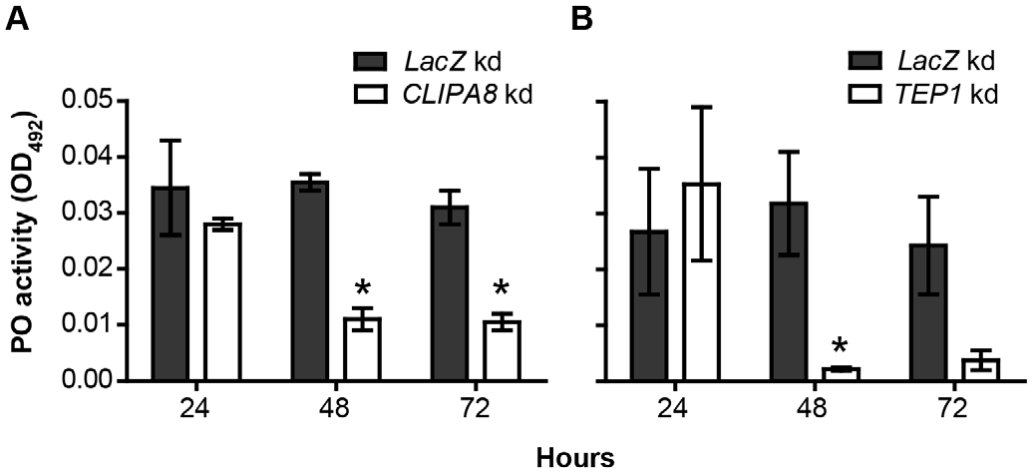
*CLIPA8* and *TEP1* kd abolished PO activity triggered during late but not early fungal infection. Phenoloxidase (PO) enzymatic activity [detected as absorbance at 492 nm, OD_492_, after conversion of L-3,4-dihydroxyphenylalanine (L-DOPA)], was measured in hemolymph extracted from (A) *CLIPA8* and (B) *TEP1* kd mosquitoes and compared to that in *LacZ* kd controls at the indicated time points after challenging mosquitoes by spraying with a *B. bassiana* (strain 80.2) suspension containing 1×10^8^ conidia/ml. The graphs show PO activity measured at 50 min after addition of L-DOPA. Means were calculated from two independent biological experiments performed using different batches of mosquitoes and fungal conidia. Error bars represent the standard error of the mean. Statistical analysis was performed using the Student's t-test and differences were considered to be statistically significant if *P*<0.05 (indicated by an asterisk).

The microscopic analysis described above indicates that the early melanotic response to conidia injection requires the direct participation of hemocytes while that triggered against growing hyphae is humoral and dependent on TEP1 and CLIPA8. To investigate further this point, we measured the temporal dynamics of hemolymph PO activity in *CLIPA8*, *TEP1* and *LacZ* kd mosquitoes at 24, 48 and 72 h after spraying with a suspension of 1×10^8^ conidia/ml. PO activities in both *CLIPA8* (Figure 4A) and *TEP1* kd mosquitoes (Figure 4B) were similar to that in *LacZ* kd controls at 24 h post-challenge, when the fungus has just invaded the cuticle. However, at later time points, the activity dropped significantly in both *CLIPA8* and *TEP1* kd mosquitoes while it remained relatively unchanged in controls, which further supports the humoral nature of the melanotic response to late fungal stages.

### PPO recruitment to hyphae is dependent on both TEP1 and CLIPA8

**Figure 5:**
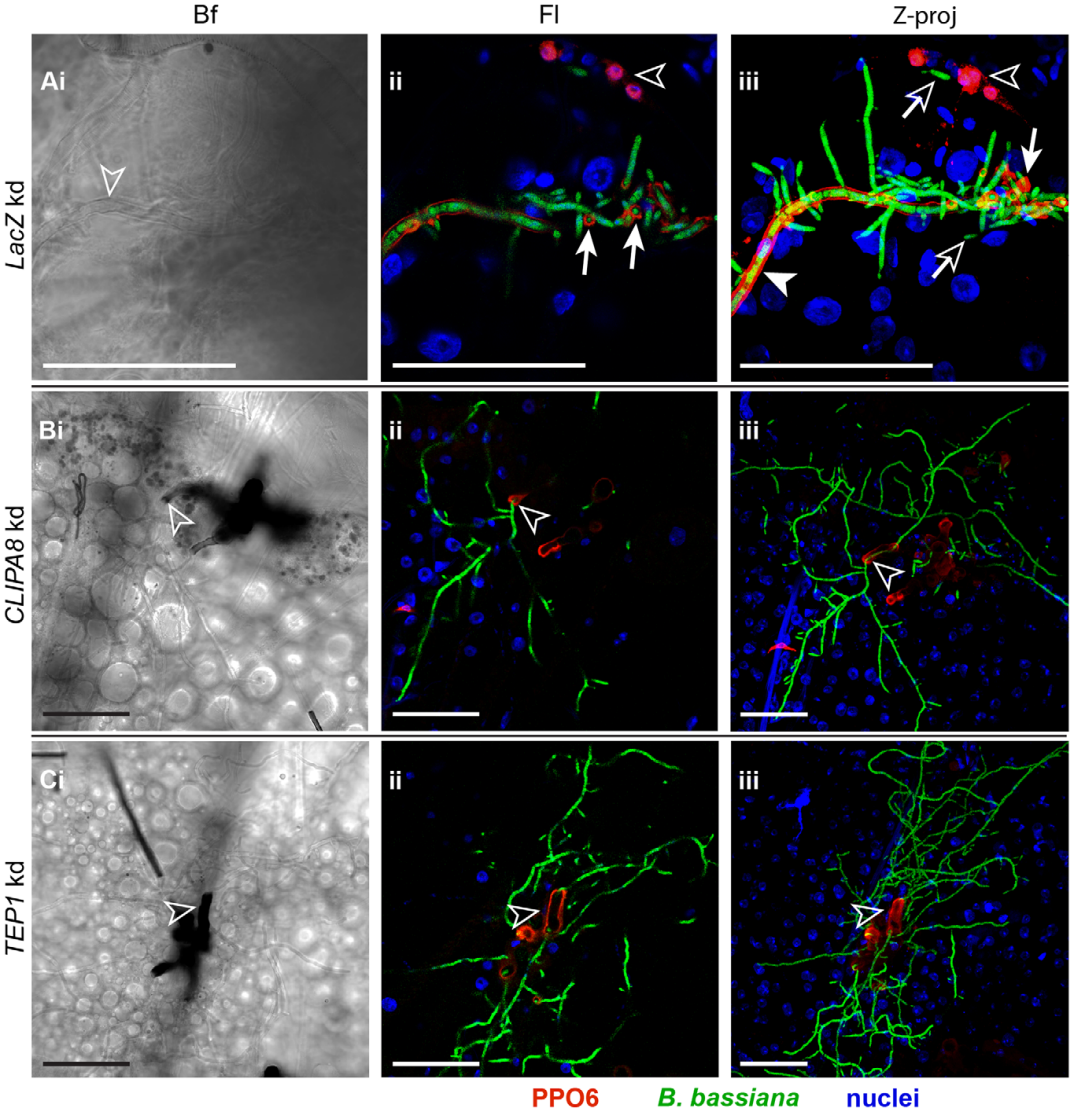
PPO recruitment to hyphae requires TEP1 and CLIPA8. Mosquito abdomens were dissected from (A) *LacZ*, (B) *CLIPA8* and (C) *TEP1* kd mosquitoes at 48 h post-injection of each with 200 conidia of GFP-expressing *B. bassiana* and stained with PPO6 antibody. Shown are Bright field (Bf) and fluorescence (Fl) images of confocal sections, and Z projections (Z-proj) of whole stacks. (A) An abdomen from *LacZ* kd mosquitoes at 100× magnification showed uniform PPO staining along an established hypha (Aiii, filled arrowhead) elaborating new hyphae and hyphal bodies. Note that melanin was barely detectable on this hyphal surface (Ai, open arrowhead) despite its intense PPO staining. A strong PPO signal was also observed at the branching points of the established hypha (Aii and Aiii, arrows with filled heads). The hyphal bodies detected were not labelled with PPO (Aii and Aiii, arrows with open heads). Shown also are PPO-expressing hemocytes (Aii and Aiii, open arrowheads). (B) *CLIPA8* and (C) *TEP1* kd mosquito abdomens at 40× magnification showing the absence of PPO and hence melanin from hyphal surfaces despite the extensive mycelial growth in these genotypes; melanin formation (Bi and Ci, open arrowheads) and PPO staining (Bii–iii and Cii–iii, open arrowheads) were restricted only to the base of the mycelium from which hyphae emerged. PPO6 (red), *B. bassiana*-GFP (green) and nuclei (blue). All scale bars are 50 µm.

Initiation of the melanization reaction requires limited proteolytic cleavage of zymogen PPO into active PO, the rate limiting enzyme in melanogenesis. The mechanisms which trigger PPO recruitment to microbial surfaces remain unclear. Here, we analyzed PPO localization to hyphae in *TEP1*, *CLIPA8* and *LacZ* kd (control) mosquitoes at 48 h after conidia injection, using confocal microscopy. In the control group, PPO staining was observed on mycelial structures coated with a thick melanin capsule (data not shown) as previously reported in Figure 3B. Additionally, PPO was also detected along the length of hyphae on which melanin deposition was barely detectable or even absent (Figure 5A), as if a lag phase existed between PPO recruitment and melanogenesis on these hyphal surfaces. PPO staining was often detected around the branching points of established hyphae (Figure 5A). Interestingly, silencing *CLIPA8* or *TEP1* completely abolished PPO localization to hyphae, and consequently none of these structures was melanized (Figures 5B and 5C, respectively). However, in these genotypes, PO was still detected on the melanized base of the mycelium from which hyphae emerged, corroborating our previous conclusion that the melanotic response against the early fungal stages is independent of TEP1 and CLIPA8 functions. Interestingly, the rare hyphal bodies detected in control mosquitoes at that time point, were not labelled with PPO (Figure 5A) suggesting that these stages might escape melanization.

**Figure 6:**
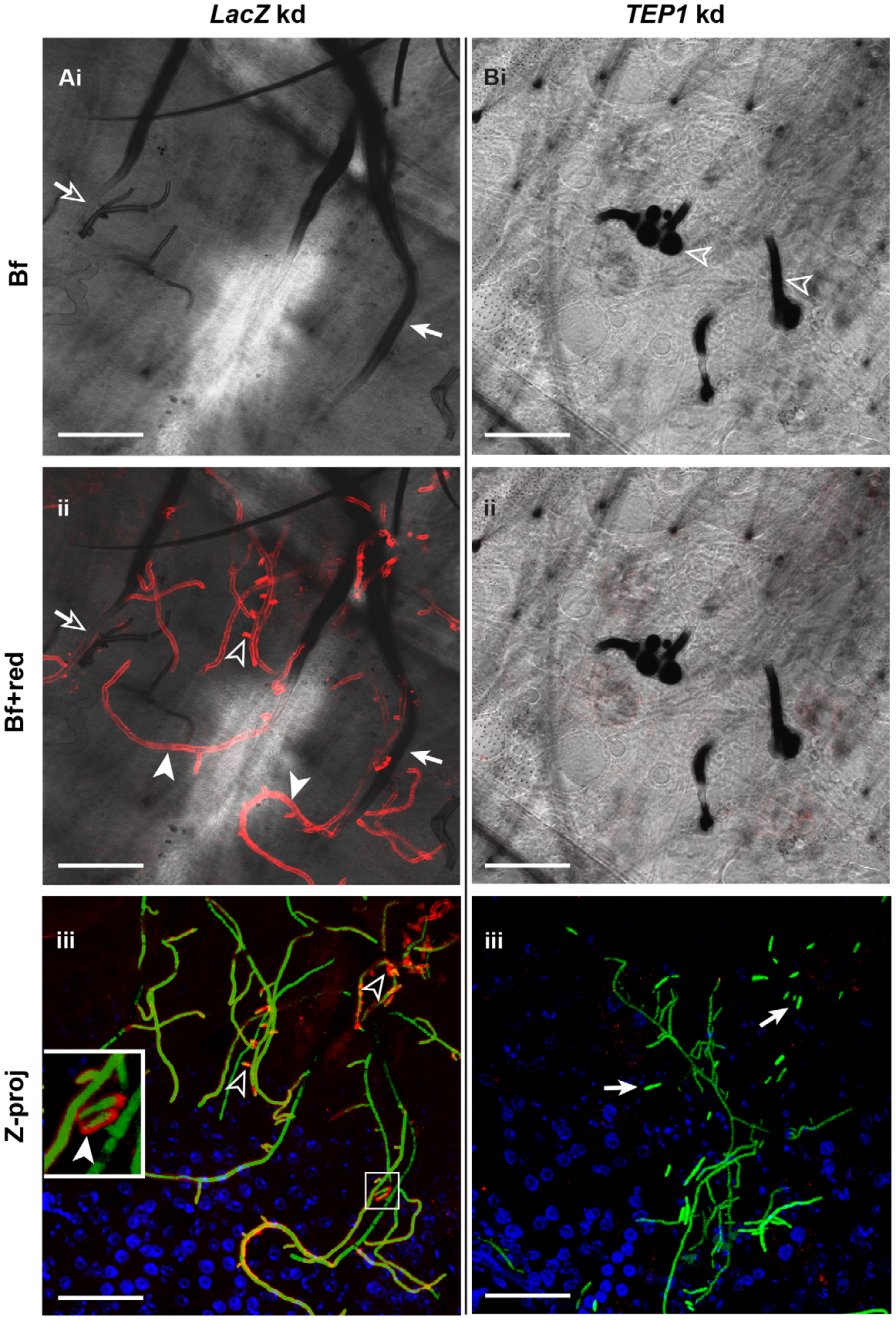
TEP1 localizes to hyphae and hyphal bodies. (A) *LacZ* (control) and (B) *TEP1* kd mosquitoes were injected each with 200 conidia of GFP-expressing *B. bassiana*. Abdomens were dissected 48 h later and immunostained with TEP1 antibody. Shown are bright field (Bf) and merged bright field and red channel (Bf+red) confocal sections and Z projections (Z-proj) of whole stacks. (A) In control mosquitoes TEP1 staining was observed on established hyphae (Aii, filled arrowheads), young branching hyphae (Aii and Aiii, open arrowheads) and hyphal bodies (Aiii, inset and filled arrowhead). Most TEP1-stained hyphae did not exhibit melanin formation at that time point (compare Ai and Aii), except in rare cases where TEP1 signal overlapped with a thin layer of melanin (Ai and Aii, arrows with open heads). TEP1 was not detected on heavily melanized hyphal surfaces probably because it was masked by melanin (Ai and Aii, arrows with filled heads). (B) TEP1 staining of hyphae was completely abolished in *TEP1* kd mosquitoes (compare Aii with Bii, and Aiii with Biii). Consequently, melanin did not form over these hyphal surfaces but was present only on early fungal stages including germinating spores and germ tubes (Bi, open arrowheads). Note the abundance of hyphal bodies in *TEP1* kd (Biii, arrows) compared to controls. TEP1 (red) GFP-expressing *B. bassiana* (green), nuclei (blue). All scale bars are 50 µm.

TEP1 binds to bacteria enhancing their phagocytosis by a hemocyte-like cell line [34] and to *Plasmodium* ookinetes, as they egress from midgut epithelial cells into the basal labyrinth, leading to their lysis. The fact that TEP1 binds to evolutionary distant microbial surfaces and that PPO localization to hyphae is TEP1-dependent, prompted us to study whether TEP1 associates with hyphal surfaces to trigger downstream events culminating in PPO activation and subsequent melanin formation. Abdomens dissected from control (*LacZ* kd) mosquitoes at 48 h after conidia injections revealed strong TEP1 localization on melanin-free hyphal surfaces (Figure 6A); where melanin had previously formed, TEP1 signal was either faint or absent, possibly because it was masked by the thick melanotic capsule. Also, the rare hyphal bodies detected in these abdomens were labelled with TEP1. Thus, TEP1 localization to hyphal surfaces clearly precedes melanin formation; the tips from where hyphae grew were always TEP1 positive but melanin negative (Figure 6A). In *TEP1* kd mosquitoes, the melanization of hyphae was completely abolished (Figure 6B). Interestingly, hyphal bodies were more common in these mosquitoes relative to controls, suggesting rapid fungal growth. In summary, our data revealed that TEP1 association with hyphal surfaces is a prerequisite for the initiation of a local melanotic reaction against *B. bassiana*.

### The mosquito melanization response protects against *B. bassiana* infection

**Figure 7:**
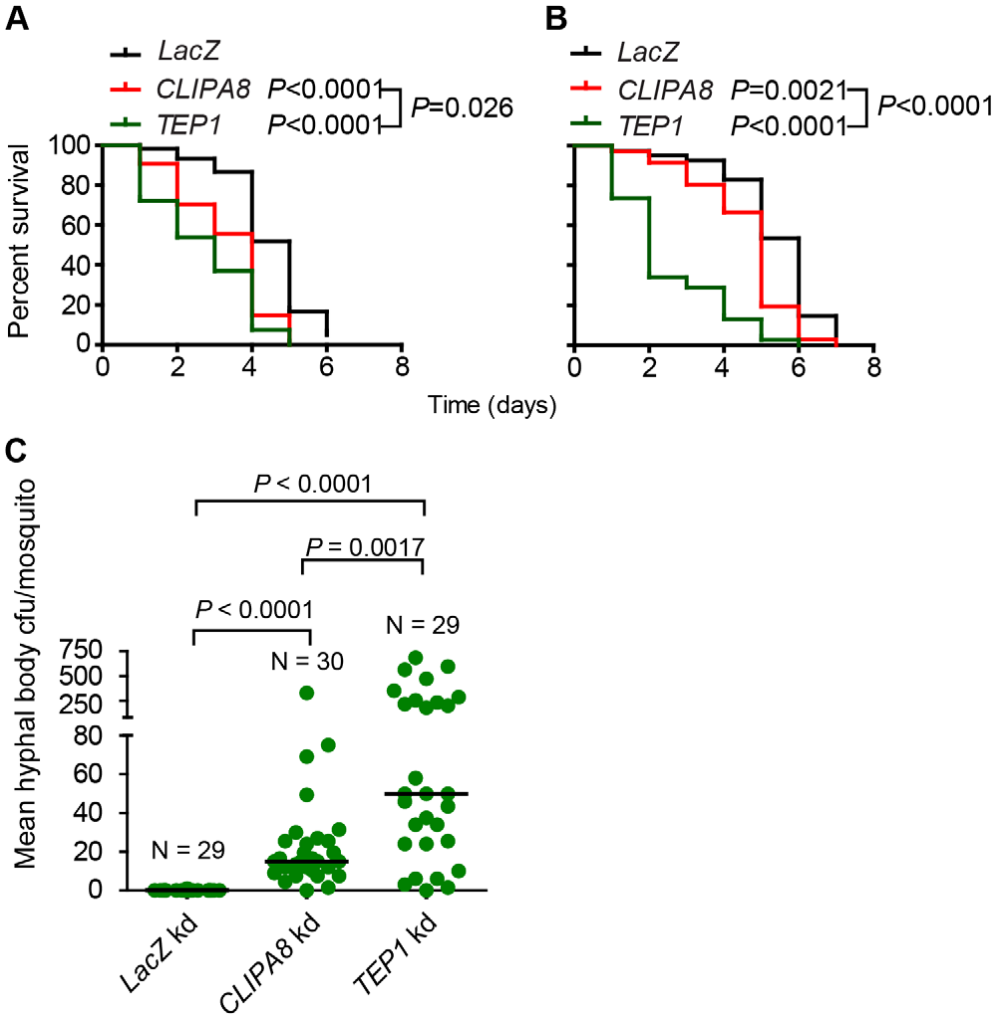
*CLIPA8* and *TEP1* kd mosquitoes are more sensitive to *B. bassiana* infections. *LacZ*, *TEP1* and *CLIPA8* kd adult female *A. gambiae* mosquitoes were challenged with *B. bassiana* (strain 80.2) either by (A) bringing the mosquitoes in contact with a lawn of conidia on fungal PDA plates or (B) by spraying mosquitoes with a suspension of 1×10^8^ conidia/ml. Dead mosquitoes were counted daily over the indicated period. Graphs represent percent survival as calculated by the Kaplan-Meier method for one representative experiment of each treatment. Statistical significance was calculated by the log rank test. Survival curves were considered to be significantly different if *P*<0.05. (C) *B. bassiana* infected *CLIPA8* and *TEP1* kd mosquitoes contained significantly more hyphal body colony forming units (cfu) than infected *LacZ* kd controls. Here, mosquitoes were challenged by spraying with *B. bassiana* strain 80.2 at a suspension of 5×10^7^ conidia/ml and batches of two mosquitoes each were collected four days later, surface sterilized, grinded and serial dilutions plated on *B. bassiana* selective medium. Each circle represents mean hyphal body cfu per mosquito per batch. Medians are indicated with horizontal lines and were 0, 15 and 50 for *LacZ*, *CLIPA8* and *TEP1* kd mosquitoes, respectively. The numbers of batches (N) processed per genotype are indicated. Statistical significance was calculated using the Mann-Whitney test; medians were considered significant if *P*<0.05. Results are from two independent biological experiments involving different batches of mosquitoes and fungal conidia.

The microscopic observation of melanotic capsules around entomopathogenic fungi has been reported earlier in several insect species including *Chironomus* [35], the leafhopper *Empoasca fabae* [36] and the grasshopper *Melanoplus sanguinipes* [37]. However, the relative contribution of this immune response to anti-fungal defense remains poorly understood. In *A. gambiae*, melanization is dispensable for defense against bacterial infections, despite the fact that bacteria trigger PPO activation in the hemolymph [9]. Additionally, field caught *A. gambiae* mosquitoes [22] as well as most laboratory strains rarely melanize *Plasmodium* ookinetes suggesting that melanization is dispensable for defense against these parasite stages. The fact that mosquitoes mounted a strong melanotic response to *B. bassiana* prompted us to test the relevance of this response to anti-fungal immunity. To address this point, *TEP1*, *CLIPA8* and *LacZ* kd adult female mosquitoes were naturally infected with a wildtype *B. bassiana* strain (80.2) either by spraying with a suspension of 1×10^8^ condia/ml or by gentle dragging over a lawn of spores on a potato dextrose agar plate. Mosquitoes were then incubated at 27°C at 90% humidity and their survival scored on a daily basis. Survival assays revealed a significant increase in susceptibility of *CLIPA8* and *TEP1* kd mosquitoes to *B. bassiana* over controls, whether gentle dragging (Figure 7A) or spraying (Figure 7B) was used to establish an infection. Interestingly, the *TEP1* kd group succumbed more quickly to infection than the *CLIPA8* kd, suggesting that TEP1 might be controlling more than one anti-fungal effector mechanism.

We then scored hyphal body colony forming units in *CLIPA8*, *TEP1* and *LacZ* kd mosquitoes four days after spraying with 5×10^7^ conidia/ml, to determine whether the compromised survival in the two former genotypes is due to increased fungal proliferation. Our data revealed that *CLIPA8* and *TEP1* kd mosquitoes contained indeed significantly higher numbers of hyphal bodies than controls which contained none at that time point; the median values were 15, 50 and 0, respectively (Figure 7C). Here, it is worth mentioning that, in general, hyphal bodies appeared more quickly in wildtype mosquitoes when fungal infection was established through injection rather than the natural route. This explains why these stages were sometimes detected in whole mounts of abdomens at 48 h post conidia injection (Figures 5A and 6B), but were absent from mosquitoes even at day four after natural infection (Figure 7C). Hyphal bodies were significantly more abundant in *TEP1* (*P* = 0.0017) than in *CLIPA8* kd mosquitoes, which explains the increased sensitivity of the former genotype to *B. bassiana* challenge.

## Discussion

Melanization is an important immune response in insects that is triggered against diverse microbial classes including parasites [38], bacteria [9], [15], [39] and fungi [35]–[37]. Functional genetic studies in several insect species revealed an important role for this response in insect immunity to bacterial infections [10], [11], [15]. Here, we investigated the role of melanization in *A. gambiae* anti-fungal defense using *B. bassiana* as a model. Our work has been prompted by early electron microscopy studies showing the formation of melanotic capsules around pathogenic fungi invading the hemocoel of other insect species [36], [37], and by the fact that entomopathogenic fungi employ a different route for mosquito invasion compared to bacteria and *Plasmodium* parasites. The latter two, naturally infect through the oral route and traverse the midgut epithelium in order to gain access into the hemocoel, whereas pathogenic fungi breach the cuticle reaching directly into the hemocoel using a combination of mechanical pressure and an array of cuticle-degrading enzymes [40].

Results obtained from temporal analysis of the melanotic response to *B. bassiana* developmental stages in adult mosquitoes, using fluorescent microscopy, are in line with early reports showing that this response did not prevent the germination of *B. bassiana* conidia in the hemolymph of other insect species [37], [41]. Melanization occurred rapidly on injected conidia, then progressed over the germ tubes as well as hyphae that constitute the bulk of the mycelium (Figure 1). Only in rare cases was the mycelium completely melanized, rather hyphae almost always succeeded to break through the melanotic capsule. Our results revealed that the mosquito mounts a potent melanotic response against the fungus, with melanized hyphae sometimes measuring more than one millimeter in length (data not shown). This response, however, is not sufficient to kill the fungus. A possible explanation could be the depletion of hemolymph PPO later during infection, due to the continuous triggering of the response by the rapidly growing fungus; however, western blot analysis excluded such possibility since PPO levels remained relatively unchanged in the hemolymph up to five days post-infection (Figure S1). Nevertheless, we provided, for the first time, tangible evidence that melanization retards significantly the growth of the fungus in the mosquito. This is reflected in the absence of hyphal bodies in control (*LacZ* kd) mosquitoes four days after spraying with a conidial suspension, compared to their presence in *CLIPA8* and *TEP1* kd mosquitoes processed at the same time (Figure 7C).

The delay in the differentiation of hyphal bodies in control mosquitoes is probably imposed by the strong melanotic response triggered against hyphae. This is supported by the detection of PPO and TEP1 staining not only on hyphae but also around the branching points where new hyphae and possibly hyphal bodies emerged (Figures 5A and 6A, respectively). Delaying or inhibiting hyphal body differentiation may limit fungal dissemination, since these single cell stages proliferate in the hemolymph ultimately establishing their own mycelia. It was previously reported that melanin exhibits anti-fungal properties *in vitro* against *Aphanomyces astaci* [42] and *Metarhizium anisopliae* [43], however, the mechanism by which it interferes with fungal growth is still not clear. A plausible explanation is the ability of melanin to bind and inhibit the activity of a wide range of proteins [44] including lytic enzymes produced by microbes, such as chitinases, which are involved in fungal cell wall remodeling during cell division [45]. Hence, melanin might slow down fungal growth by interfering with the synthesis of new cell wall material during that process.

The rare hyphal bodies detected in control mosquitoes at 48 h after conidia injection were not melanized nor exhibited a PPO signal (Figure 5A), suggesting that they escape melanization. The evasion of host defense by these *in vivo* stages have been proposed earlier and was attributed to their minimal cell wall which lacks immuno-stimulatory carbohydrates [29]. A more recent study based on lectin-mapping revealed that *B. bassiana* developmental stages exhibit differences in the composition of surface carbohydrates, in particular hyphal bodies which seem to shed most carbohydrate epitopes from their surface [30]. This minimal cell wall, however, did not prevent TEP1 association with the surface of hyphal bodies (Figure 6A). TEP1 recruitment to GFP-expressing *P. berghei* ookinetes triggers parasite lysis as reflected by the loss of cytoplasmic GFP signal and membrane blebbing [16]. TEP1 labelled hyphal bodies were still expressing GFP and did not show an aberrant morphology, however, it is difficult to conclude at that stage whether they are live or not without detailed electron microscopy analysis. Nevertheless, the fact *TEP1* kd mosquitoes exhibited significantly higher numbers of hyphal bodies and increased sensitivity to natural *B. bassiana* infections compared to *CLIPA8* kd, inform an additional, melanization-independent role of TEP1 in limiting fungal growth, that remain to be defined. TEP1 is known to be an important anti-bacterial factor [34], [46], which raises the possibility that the rapid death observed in fungal infected *TEP1* kd mosquitoes could be due to the proliferation of opportunistic bacterial infections rather than fungal proliferation. However, this possibility was excluded because fungal infection triggered a similar survival pattern in *TEP1* kd mosquitoes pre-treated with a cocktail of antibiotics (Figure S2).

Using immunohistochemistry and confocal microscopy, we observed a PPO-positive signal on hyphae that exhibited either minimal or no melanin formation (at least within the resolving power of light microscopy) in control mosquitoes (Figure 5A). On these hyphae melanogenesis appeared lagging behind fungal growth, whereby apical parts of hyphae exhibited strong PPO staining but minimal or no melanin formation, while the basal parts showed PPO staining around thick melanotic capsules. This is the first time mosquito PPO is detected on microbial surfaces that do not exhibit clear signs of melanin formation. In *A. gambiae*-*P. berghei* model system, a PPO signal was always detected around dead parasites confined in a dense melanotic capsule [16]. A plausible explanation for this unusual pattern of PPO localization to hyphae is that the rapidly growing fungus probably exhausts the mosquito melanotic response. This is supported by the finding that PO activity in control mosquitoes did not change significantly between 24 and 72 h post infection, suggesting continuous PPO activation, at least during that period (Figure 4).

The depletion of TEP1 or CLIPA8 from mosquito hemolymph completely abolished PPO localization to hyphae and their subsequent melanization, suggesting that PPO recruitment to fungal surfaces is an indirect process, that depends, most likely, on the prior assembly of an immune protein complex on microbial surfaces. Whether this complex includes TEP1 and CLIPA8, and whether these two proteins recruit PPO directly or indirectly to microbial surfaces remain to be elucidated. Our data also revealed that, in addition to their role as cofactors for PPO activation [6], [47], clip-domain serine protease homologs seem to be required for PPO recruitment to microbial surfaces. It was previously reported that TEP1 binding to ookinetes in a melanotic refractory strain of *A. gambiae*, triggered their lysis and subsequent melanotic encapsulation. In that study, the authors proposed that PPO activation and recruitment was triggered by dead parasites, already killed by TEP1, and not by TEP1 itself, suggesting that the melanotic response is reminiscent of wound healing and does not represent an immune defense reaction *per se* [16]. It is difficult to reconcile our findings with those of the above study for the following reasons. First, in our infection model, PPO does not seem to be recruited to the surface of a dying fungus since melanized hyphae were not killed and were still growing at their tips, often elaborating lateral branches. Second, even though we did not assay directly the co-localization of TEP1 and PO (both antibodies were produced in the same host species), the fact that both were able to bind hyphae exhibiting minimal or no melanin formation, suggests that they might co-localize on hyphal surfaces.

The *A. gambiae* melanization response can be triggered by bacteria [9], *Plasmodium* ookinetes [17], [48], Sephadex beads [49] and fungi (according to this report). Further, certain immune proteins like TEP1 ([16], [49] and unpublished data) and CLIPA8 [8], [9] are required in all these melanotic events; of note, the role of CLIPA8 in bead melanization has not been addressed but is expected to be also essential. Altogether, these findings indicate that the molecular mechanisms that underlie the mosquito melanization response to foreign bodies with distinct biochemical surface characteristics are controlled to a certain extent by the same genetic loci. They also raise intriguing questions as to the nature of the upstream molecular recognition process that triggers the melanotic response to each of these foreign surfaces, especially that Sephadex beads, which are inanimate bodies, are still efficiently melanized in the hemolymph.

In this report, we also showed that the mosquito elicits both cellular and humoral melanotic responses against *B. bassiana*. The fact that neither *TEP1* nor *CLIPA8* kd abolished melanization of the early fungal stages (Figures 2 and 6B) despite being essential proteins in this response [8], [9], [16], and the detection of a layer of hemocytes around germinating spores (Figure 3A), suggested an early cellular melanotic response. These findings challenge the current belief that melanization in insect stages with limited numbers of hemocytes, such as adult mosquitoes, occurs in a humoral manner without the direct participation of hemocytes [50]. It is possible that these cellular responses were missed because they are rare events elicited in specific cases against particular pathogens. Surprisingly, no hemocytes were observed close to hyphae later during infection, indicating that the melanotic response to these stages is humoral. There are two plausible explanations for this phenomenon which are not necessarily mutually exclusive. First, lectin mapping assays revealed that different developmental stages of *B. bassiana* display diverse surface carbohydrates [30]. Since sugars play important roles in non-self recognition, distinct sugar signatures may elicit different immune responses. Second, *B. bassiana* may interfere, at some point during its development, with the migration of hemocytes, as previously reported in the larvae of *Spodoptera exigua* infected with this fungus [51]. We would like to point out however, that the melanotic response elicited against hyphae, although humoral in nature, still depends on an indirect role of hemocytes being the main producers of many immunity proteins including TEP1 [34] PPO and CLIPA8 [33].

In summary, the interactions between the mosquito melanotic response and *B. bassiana* are evocative of an “arms race” where the fungus is almost always the winner. This does not mean that melanization is not protective; we have provided evidence that this immune response retards significantly fungal growth, and might severely compromise or even completely abrogate the growth of fungi that are less virulent than *B. bassiana*. In fact, by successfully adapting to a particularly wide host range, *B. bassiana* must have evolved strategies to overcome insect immune defenses and enhance its pathogenesis. This is supported by recent insights from the *B. bassiana* genome which revealed species-specific expansions of gene families encoding toxins, proteases and putative effector proteins that may be associated with *B. bassiana* host flexibility and pathogenesis [52].

Based on our findings, we propose that transgenic *B. bassiana* strains designed to incapacitate the mosquito melanotic response once in the hemolymph may prove to be more potent biocontrol agents than wildtype strains. Finally, the observed delay between PPO localization to hyphal surfaces and melanogenesis renders *B. bassiana* a tractable model to study the yet poorly understood molecular interactions that culminate in PPO tethering and activation on microbial surfaces; these studies would be difficult to perform in other infection models where the microbe becomes quickly melanized upon contact with the hemolymph, as in the case of *Plasmodium* ookinetes.

## Materials and Methods

### Ethics statement

This study was carried in accordance with the recommendations in the Guide for the Care and Use of Laboratory Animals of the National Institutes of Health, U.S.A. The Institutional Animal Care and Use Committee (IACUC) of the American University of Beirut approved the animal protocol (permit number 11-09-199). The IACUC functions in compliance with the Public Health Service Policy on the Humane Care and Use of Laboratory Animals (USA), and adopts the Guide for the Care and Use of Laboratory Animals of the National Institutes of Health, U.S.A.

### *Anopheles gambiae* rearing, antibiotic treatment, and *Beauveria bassiana* strains

All experiments were performed with *Anopheles gambiae* G3 strain which was reared as previously described [53]. Briefly, *A. gambiae* mosquitoes were maintained at 27°C and 80% humidity with a 12 h day-night cycle. Larvae were reared on tropical fish food. Adult mosquitoes were maintained on 10% sucrose and given mice blood (Mice were anaesthetized with ketamine) for egg production. For antibiotic treatment, freshly emerged mosquitoes were maintained on a 10% sucrose solution containing gentamycin (15 µg/ml), penicillin (10 units/ml) and streptomycin (10 µg/ml) for six days prior to infection with *B. bassiana*, and isolated from the rest of the colony in closed plastic containers. Fresh antibiotics solution was provided every 12 h. This antibiotic regimen was continued for an additional 24 h after fungal infection then stopped. Wildtype *B. bassiana* strain 80.2 (a kind gift of D. Ferrandon) and a GFP-expressing strain (242-GFP; a kind gift of M. Bidochka) were cultured on potato dextrose agar (PDA) plates at 25°C and 80% humidity. Conidia (spores) used for mosquito challenges were harvested from 3–4 weeks old cultures by adding 10 ml ddH_2_O to each PDA plate and scraping the surface of the mycelia with sterile cell scrapers. Conidia were separated from other mycelial structures over a sterile funnel packed with autoclaved glass wool, washed two times with ddH_2_O by centrifugation at 4000 rpm, counted and diluted to the appropriate concentration. Freshly prepared conidia were used for all experiments.

### Gene silencing by RNA interference

Double-stranded RNAs (dsRNA) for *LacZ* (control), *TEP1* and *CLIPA8* were synthesized as previously described [9], [16], [21], respectively. *In vivo* gene silencing by RNA interference was performed as previously reported [31] and efficiency of gene silencing was confirmed by immunoblotting.

### Immunoblotting

Hemolymph proteins were extracted from *LacZ*, *CLIPA8* and *TEP1* kd mosquitoes by proboscis clipping directly into 1× non reducing Lane Marker Sample Buffer (Pierce), separated on 8% SDS-PAGE, then transferred to Immun-Blot PVDF membrane (BioRad) by semi-dry blotting (BioRad). Membranes were probed with rabbit polyclonal α-TEP1 (1/1000), mouse monoclonal α-CLIPA8 (1/30), rabbit polyclonal α-SRPN3 (1/1000) and rabbit polyclonal α-PPO6 (1/1000) [32]. The latter two antibodies served as loading controls. Horse raddish peroxidase-conjugated α-mouse and α-rabbit secondary antibodies were used at 1/5000 and 1/12000, respectively. To determine hemolymph PPO levels after fungal infection, mosquitoes were sprayed with a suspension of *B. bassiana* (strain 80.2) containing 1×10^8^ conidia/ml in 0.05% Tween-80, and hemolymph was collected from 20 mosquitoes at each of the indicated time points using the same procedure describe above. Rabbit polyclonal α-TEP1 and horse raddish peroxidase-conjugated α-rabbit secondary antibodies were used at (1/1000) and (1/12000), respectively.

### Mosquito survival and *B. bassiana* proliferation assay

Freshly emerged adult mosquito females were injected with dsRNAs (3 µg/µl) for *LacZ*, *TEP1* and *CLIPA8*, left four days to recover, then challenged with *B. bassiana* (strain 80.2). For survival assays, fungal challenges were conducted in two ways. A batch of fifty cold-anaesthetized mosquitoes per genotype were either sprayed with a suspension of 1×10^8^ conidia/ml in 0.05% Tween-80, using glass atomizers purchased from sally@AccessoriesforFragrances.com, or dragged gently over a lawn of conidia in PDA cultures. Mosquito survival was scored on daily basis over a week. Three biological experiments were performed for each treatment. The Kaplan-Meier survival test was used to calculate the percent survival. Statistical significance of the observed differences was calculated using the Log-rank test.

For the fungal proliferation assay, *LacZ*, *TEP1* and *CLIPA8*-silenced mosquitoes were sprayed with a suspension of *B. bassiana* (strain 80.2) containing 5×10^7^ conidia/ml in 0.05% Tween-80. Four days post-infection, approximately 15 batches of 2 mosquitoes each per genotype were grinded in 400 µl ddH_2_O containing 0.05% Tween-80, then 50 µl of the homogenate was spread on *B. bassiana* selective medium [PDA containing 1 mg/ml yeast, 50 µg/ml Gentamycin, 50 µg/ml Penicillin, 50 µg/ml Streptomycin, 5 µg/ml Crystal violet and 250 µg/ml Dodine (Sigma)]. Plates were incubated at 25°C at 80% humidity and hyphal body colony forming units were scored six days later. The experiment was repeated twice. Statistical significance was calculated using the Mann-Whitney test; medians were considered significantly different if *P*<0.05.

### Immunohistochemistry and confocal microscopy

Four days post-gene silencing, individual mosquitoes were injected with approximately 200 conidia of GFP-expressing *B. bassiana* (strain 242-GFP). Abdomens were dissected at the indicated time points, fixed in 4% formaldehyde for 50 minutes, washed 3 times in 1× phosphate buffered saline (PBS) and blocked for 1 h at room temperature in blocking buffer (1×PBS containing 2% BSA and 0.05% Triton X-100). Then, abdomens were incubated overnight at 4°C with rabbit polyclonal α-TEP1 (1/350) or rabbit polyclonal α-PPO6 (1/500) diluted in blocking buffer. Following incubation, abdomens were washed three times with 1× PBS containing 0.05% Triton X-100, then incubated with Alexa-546 conjugated α-rabbit secondary antibody (Molecular Probes) diluted 1/800 in blocking buffer. After washing, nuclei were stained with Hoechst (1/10000) and abdomens mounted in Vectashield mounting medium (Vector Laboratories). Images were collected using on a Zeiss LSM 710 META confocal microscope.

### PPO enzymatic assay

*CLIPA8*, *TEP1* and *LacZ* kd mosquitoes were sprayed with a suspension of 1×10^8^ conidia/ml of *B. bassiana* strain 80.2. Hemolymph was collected at 24, 48 and 72 h after challenge in ice-cold phosphate buffered saline (PBS) containing protease inhibitors and protein concentration was determined using the Bradford Reagent (Fermentas). PO enzymatic assay was performed as previously described [9] using approximately 5 µg hemolymph proteins per reaction. Absorbance at 492 nm was measured in a Multiskan Ex microplate reader (ThermoLabsystems) after incubation with L-DOPA at room temperature for 50 min.

## Supporting Information

Figure S1 **Hemolymph PPO levels after natural** ***B. bassiana*** **infection.** Western blot analysis showing hemolymph PPO levels in adult female mosquitoes at the indicated times points after spraying them with a suspension of 1×10^8^ conidia/ml of *B. bassiana* (strain 80.2). Each lane contains hemolymph extracts from 20 mosquitoes. C, control non-infected mosquitoes. Asterisks indicate non-specific bands.
(TIF)

Figure S2 ***TEP1*** **-silenced aseptic mosquitoes are still sensitive to** ***B. bassiana*** **infection.** *LacZ* and *TEP1* kd female *A. gambiae* mosquitoes treated with antibiotics, to eliminate or at least reduce substantially their microbial flora, were challenged with *B. bassiana* (strain 80.2) by spraying mosquitoes with a suspension of 1×10^8^ conidia/ml. Dead mosquitoes were counted daily over the indicated period. (A) and (B) Two independent experiments performed with different batches of mosquitoes and fungal conidia. Graphs represent percent survival as calculated by the Kaplan-Meier method. Statistical significance was calculated by the log rank test. Survival curves were considered to be significantly different if *P*<0.05.
(TIF)

## References

[1] Cerenius L, Lee BL, Soderhall K (2008) The proPO-system: pros and cons for its role in invertebrate immunity. Trends Immunol 29: 263–271. 18457993 10.1016/j.it.2008.02.009

[2] Park JW, Kim CH, Kim JH, Je BR, Roh KB, et al (2007) Clustering of peptidoglycan recognition protein-SA is required for sensing lysine-type peptidoglycan in insects. Proc Natl Acad Sci U S A 104: 6602–6607. 17409189 10.1073/pnas.0610924104 PMC1871832

[3] Wang Y, Jiang H (2006) Interaction of beta-1,3-glucan with its recognition protein activates hemolymph proteinase 14, an initiation enzyme of the prophenoloxidase activation system in Manduca sexta. J Biol Chem 281: 9271–9278. 16461344 10.1074/jbc.M513797200 PMC2020818

[4] Levashina EA, Langley E, Green C, Gubb D, Ashburner M, et al (1999) Constitutive activation of toll-mediated antifungal defense in serpin-deficient *Drosophila* . Science 285: 1917–1919. 10489372 10.1126/science.285.5435.1917

[5] Michel K, Budd A, Pinto S, Gibson TJ, Kafatos FC (2005) *Anopheles gambiae* SRPN2 facilitates midgut invasion by the malaria parasite *Plasmodium berghei* . EMBO Rep 6: 891–897. 16113656 10.1038/sj.embor.7400478 PMC1369158

[6] Yu XQ, Jiang H, Wang Y, Kanost MR (2003) Nonproteolytic serine proteinase homologs are involved in prophenoloxidase activation in the tobacco hornworm, *Manduca sexta* . Insect Biochem Mol Biol 33: 197–208. 12535678 10.1016/s0965-1748(02)00191-1

[7] Lee KY, Zhang R, Kim MS, Park JW, Park HY, et al (2002) A zymogen form of masquerade-like serine proteinase homologue is cleaved during pro-phenoloxidase activation by Ca2+ in coleopteran and *Tenebrio molitor* larvae. Eur J Biochem 269: 4375–4383. 12199717 10.1046/j.1432-1033.2002.03155.x

[8] Volz J, Muller HM, Zdanowicz A, Kafatos FC, Osta MA (2006) A genetic module regulates the melanization response of *Anopheles* to *Plasmodium* . Cell Microbiol 8: 1392–1405. 16922859 10.1111/j.1462-5822.2006.00718.x

[9] Schnitger AK, Kafatos FC, Osta MA (2007) The Melanization Reaction Is not Required for Survival of *Anopheles gambiae* Mosquitoes after bacterial Infections. J Biol Chem 282: 21884–21888. 17537726 10.1074/jbc.M701635200

[10] Liu H, Jiravanichpaisal P, Cerenius L, Lee BL, Soderhall I, et al (2007) Phenoloxidase is an important component of the defense against *Aeromonas hydrophila* Infection in a crustacean, *Pacifastacus leniusculus* . J Biol Chem 282: 33593–33598. 17855335 10.1074/jbc.M706113200

[11] Eleftherianos I, Boundy S, Joyce SA, Aslam S, Marshall JW, et al (2007) An antibiotic produced by an insect-pathogenic bacterium suppresses host defenses through phenoloxidase inhibition. Proc Natl Acad Sci U S A 104: 2419–2424. 17284598 10.1073/pnas.0610525104 PMC1892976

[12] Lu Z, Beck MH, Wang Y, Jiang H, Strand MR (2008) The viral protein Egf1.0 is a dual activity inhibitor of prophenoloxidase-activating proteinases 1 and 3 from *Manduca sexta* . J Biol Chem 283: 21325–21333. 18519564 10.1074/jbc.M801593200 PMC2490783

[13] Leclerc V, Pelte N, El Chamy L, Martinelli C, Ligoxygakis P, et al (2006) Prophenoloxidase activation is not required for survival to microbial infections in *Drosophila* . EMBO Rep 7: 231–235. 16322759 10.1038/sj.embor.7400592 PMC1369246

[14] Tang H, Kambris Z, Lemaitre B, Hashimoto C (2006) Two proteases defining a melanization cascade in the immune system of *Drosophila* . J Biol Chem 281: 28097–28104. 16861233 10.1074/jbc.M601642200

[15] Ayres JS, Schneider DS (2008) A signaling protease required for melanization in Drosophila affects resistance and tolerance of infections. PLoS Biol 6: 2764–2773. 19071960 10.1371/journal.pbio.0060305 PMC2596860

[16] Blandin S, Shiao SH, Moita LF, Janse CJ, Waters AP, et al (2004) Complement-like protein TEP1 is a determinant of vectorial capacity in the malaria vector *Anopheles gambiae* . Cell 116: 661–670. 15006349 10.1016/s0092-8674(04)00173-4

[17] Osta MA, Christophides GK, Kafatos FC (2004) Effects of mosquito genes on *Plasmodium* development. Science 303: 2030–2032. 15044804 10.1126/science.1091789

[18] Riehle MM, Markianos K, Niare O, Xu J, Li J, et al (2006) Natural malaria infection in *Anopheles gambiae* is regulated by a single genomic control region. Science 312: 577–579. 16645095 10.1126/science.1124153

[19] Riehle MM, Xu J, Lazzaro BP, Rottschaefer SM, Coulibaly B, et al (2008) Anopheles gambiae APL1 is a family of variable LRR proteins required for Rel1-mediated protection from the malaria parasite, *Plasmodium berghei* . PLoS One 3: e3672. 18989366 10.1371/journal.pone.0003672 PMC2577063

[20] Fraiture M, Baxter RH, Steinert S, Chelliah Y, Frolet C, et al (2009) Two mosquito LRR proteins function as complement control factors in the TEP1-mediated killing of *Plasmodium* . Cell Host Microbe 5: 273–284. 19286136 10.1016/j.chom.2009.01.005

[21] Povelones M, Waterhouse RM, Kafatos FC, Christophides GK (2009) Leucine-rich repeat protein complex activates mosquito complement in defense against *Plasmodium* parasites. Science 324: 258–261. 19264986 10.1126/science.1171400 PMC2790318

[22] Niare O, Markianos K, Volz J, Oduol F, Toure A, et al (2002) Genetic loci affecting resistance to human malaria parasites in a West African mosquito vector population. Science 298: 213–216. 12364806 10.1126/science.1073420

[23] Yassine H, Osta MA (2010) Anopheles gambiae innate immunity. Cell Microbiol 12: 1–9. 19804484 10.1111/j.1462-5822.2009.01388.x

[24] Sessions OM, Barrows NJ, Souza-Neto JA, Robinson TJ, Hershey CL, et al (2009) Discovery of insect and human dengue virus host factors. Nature 458: 1047–1050. 19396146 10.1038/nature07967 PMC3462662

[25] Souza-Neto JA, Sim S, Dimopoulos G (2009) An evolutionary conserved function of the JAK-STAT pathway in anti-dengue defense. Proc Natl Acad Sci U S A 106: 17841–17846. 19805194 10.1073/pnas.0905006106 PMC2764916

[26] Waldock J, Olson KE, Christophides GK (2012) *Anopheles gambiae* antiviral immune response to systemic O'nyong-nyong infection. PLoS Negl Trop Dis 6: e1565. 22428080 10.1371/journal.pntd.0001565 PMC3302841

[27] Oliveira Gde A, Lieberman J, Barillas-Mury C (2012) Epithelial nitration by a peroxidase/NOX5 system mediates mosquito antiplasmodial immunity. Science 335: 856–859. 22282475 10.1126/science.1209678 PMC3444286

[28] Bidochka MJ, Clark DC, Lewis MW, Keyhani NO (2010) Could insect phagocytic avoidance by entomogenous fungi have evolved via selection against soil amoeboid predators? Microbiology 156: 2164–2171. 20338910 10.1099/mic.0.038216-0

[29] Pendland JC, Hung SY, Boucias DG (1993) Evasion of host defense by in vivo-produced protoplast-like cells of the insect mycopathogen Beauveria bassiana. J Bacteriol 175: 5962–5969. 8376342 10.1128/jb.175.18.5962-5969.1993 PMC206677

[30] Wanchoo A, Lewis MW, Keyhani NO (2009) Lectin mapping reveals stage-specific display of surface carbohydrates in in vitro and haemolymph-derived cells of the entomopathogenic fungus Beauveria bassiana. Microbiology 155: 3121–3133. 19608611 10.1099/mic.0.029157-0

[31] Blandin S, Moita LF, Kocher T, Wilm M, Kafatos FC, et al (2002) Reverse genetics in the mosquito *Anopheles gambiae*: targeted disruption of the Defensin gene. EMBO Rep 3: 852–856. 12189180 10.1093/embo-reports/kvf180 PMC1084233

[32] Muller HM, Dimopoulos G, Blass C, Kafatos FC (1999) A hemocyte-like cell line established from the malaria vector *Anopheles gambiae* expresses six prophenoloxidase genes. J Biol Chem 274: 11727–11735. 10206988 10.1074/jbc.274.17.11727

[33] Pinto SB, Lombardo F, Koutsos AC, Waterhouse RM, McKay K, et al (2009) Discovery of Plasmodium modulators by genome-wide analysis of circulating hemocytes in Anopheles gambiae. Proc Natl Acad Sci U S A 106: 21270–21275. 19940242 10.1073/pnas.0909463106 PMC2783009

[34] Levashina EA, Moita LF, Blandin S, Vriend G, Lagueux M, et al (2001) Conserved role of a complement-like protein in phagocytosis revealed by dsRNA knockout in cultured cells of the mosquito, *Anopheles gambiae* . Cell 104: 709–718. 11257225 10.1016/s0092-8674(01)00267-7

[35] Gotz P, Enderlein G, Roettgen I (1987) Immune reactions of *Chironomus* larvae (Insecta: Diptera) against bacteria. J Insect Physiol 33: 993–1004.

[36] Butt TM, Wraight SP, Galaini-Wraight S, Humber RA, Roberts DW, et al (1988) Humoral encapsulation of the fungus *Erynia radicans* (Entomophthorales) by the potato leafhopper, *Empoasca fabae* (Homoptera: Cicadellidae). J Invertebr Pathol 52: 49–56.

[37] Bidochka MJ, Khachatourians GG (1987) Hemocytic defense response to the entomopathogenic fungus *Beauveria bassiana* in the migratory grasshopper *Melanoplus sanguinipes* . Entomol Exp Appl 45: 151–156.

[38] Li J, Tracy JW, Christensen BM (1992) Phenol oxidase activity in hemolymph compartments of *Aedes aegypti* during melanotic encapsulation reactions against microfilariae. Dev Comp Immunol 16: 41–48. 1618354 10.1016/0145-305x(92)90050-m

[39] Hillyer JF, Schmidt SL, Christensen BM (2004) The antibacterial innate immune response by the mosquito *Aedes aegypti* is mediated by hemocytes and independent of Gram type and pathogenicity. Microbes Infect 6: 448–459. 15109959 10.1016/j.micinf.2004.01.005

[40] St. Leger RJ, Charnley AK, Cooper RM (1986) Cuticle degrading enzymes of entomopathogenic fungi; mechanisms of interaction between pathogen enzymes and insect cuticle. J Invertebr Pathol 47: 295–302.

[41] Gotz P, Vey A (1974) Humoral encapsulation in Diptera (Insecta): defence reactions of *Chironomus* larvae against fungi. Parasitology 68: 193–205. 4857041

[42] Soderhall K, Ajaxon R (1982) Effect of quinone and melanin on mycelial growth of Aphanomyces spp. and extracellular protease of Aphanomyces astaci, a parasite of crayfish. J Invertebr Pathol 39: 105–109.

[43] St. Leger RJ, Cooper RM, Charnley AK (1988) The effect of melanization of Manduca sexta cuticle on growth and infection by Metarhizium anisopliae. J Invertebr Pathol 52: 459–470.

[44] Doering TL, Nosanchuk JD, Roberts WK, Casadevall A (1999) Melanin as a potential cryptococcal defence against microbicidal proteins. Med Mycol 37: 175–181. 10421849

[45] Kuo MJ, Alexander M (1967) Inhibition of the lysis of fungi by melanins. J Bacteriol 94: 624–629. 6035264 10.1128/jb.94.3.624-629.1967 PMC251933

[46] Dong Y, Aguilar R, Xi Z, Warr E, Mongin E, et al (2006) *Anopheles gambiae* immune responses to human and rodent *Plasmodium* parasite species. PLoS Pathog 2: e52. 16789837 10.1371/journal.ppat.0020052 PMC1475661

[47] Piao S, Song YL, Kim JH, Park SY, Park JW, et al (2005) Crystal structure of a clip-domain serine protease and functional roles of the clip domains. Embo J 24: 4404–4414. 16362048 10.1038/sj.emboj.7600891 PMC1356332

[48] Collins FH, Sakai RK, Vernick KD, Paskewitz S, Seeley DC, et al (1986) Genetic selection of a *Plasmodium*-refractory strain of the malaria vector *Anopheles gambiae* . Science 234: 607–610. 3532325 10.1126/science.3532325

[49] Warr E, Lambrechts L, Koella JC, Bourgouin C, Dimopoulos G (2006) Anopheles gambiae immune responses to Sephadex beads: involvement of anti-Plasmodium factors in regulating melanization. Insect Biochem Mol Biol 36: 769–778. 17027843 10.1016/j.ibmb.2006.07.006

[50] Goetz P, Roettgen I, Lingg W (1977) Encapsulement humoral en tant que reaction de defense chez les Dipteres. Ann Parasitol 52: 95–97. 900784

[51] Hung SY, Boucias DG, Vey AJ (1993) Effect of *Beauveria bassiana* and *Candida albicans* on the cellular defense response of *Spodoptera exigua* . J Invertebr Pathol 61: 179–187. 8463710 10.1006/jipa.1993.1032

[52] Xiao G, Ying SH, Zheng P, Wang ZL, Zhang S, et al (2012) Genomic perspectives on the evolution of fungal entomopathogenicity in *Beauveria bassiana* . Sci Rep 2: 483. 22761991 10.1038/srep00483 PMC3387728

[53] Danielli A, Loukeris TG, Lagueux M, Muller HM, Richman A, et al (2000) A modular chitin-binding protease associated with hemocytes and hemolymph in the mosquito Anopheles gambiae. Proc Natl Acad Sci U S A 97: 7136–7141. 10860981 10.1073/pnas.97.13.7136 PMC16512

