# Supplementary figures and images for: The Mosquito Melanization Response Is Implicated in Defense against the Entomopathogenic Fungus Beauveria bassiana
Source: PLoS Pathog. 2012 Nov 15;8(11):e1003029. doi: 10.1371/journal.ppat.1003029 (PMC3499577; doi:10.1371/journal.ppat.1003029)

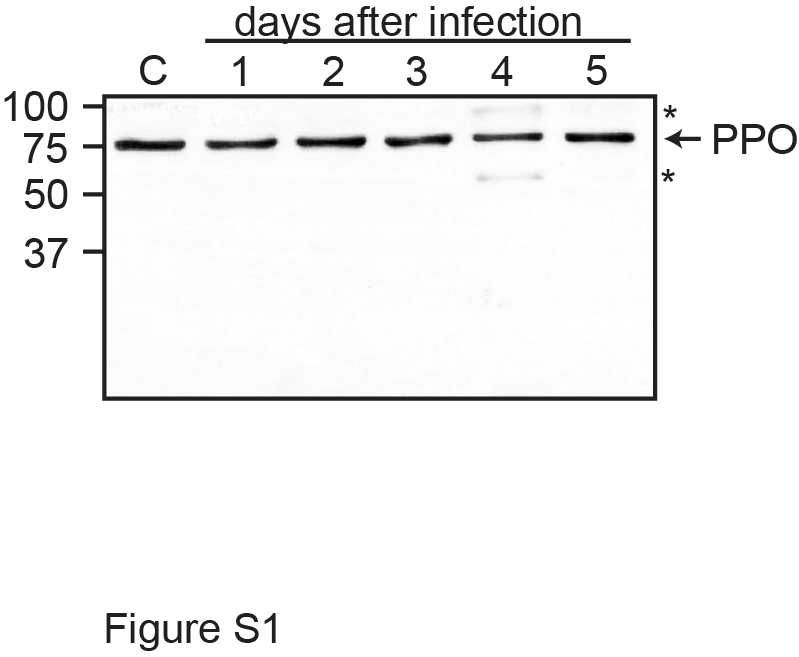

Supplement: Figure S1 — Hemolymph PPO levels after natural B. bassiana infection. Western blot analysis showing hemolymph PPO levels in adult female mosquitoes at the indicated times points after spraying them with a suspension of 1×10^8^ conidia/ml of B. bassiana (strain 80.2). Each lane contains hemolymph extracts from 20 mosquitoes. C, control non-infected mosquitoes. Asterisks indicate non-specific bands. (TIF) [file ppat.1003029.s001.tif]

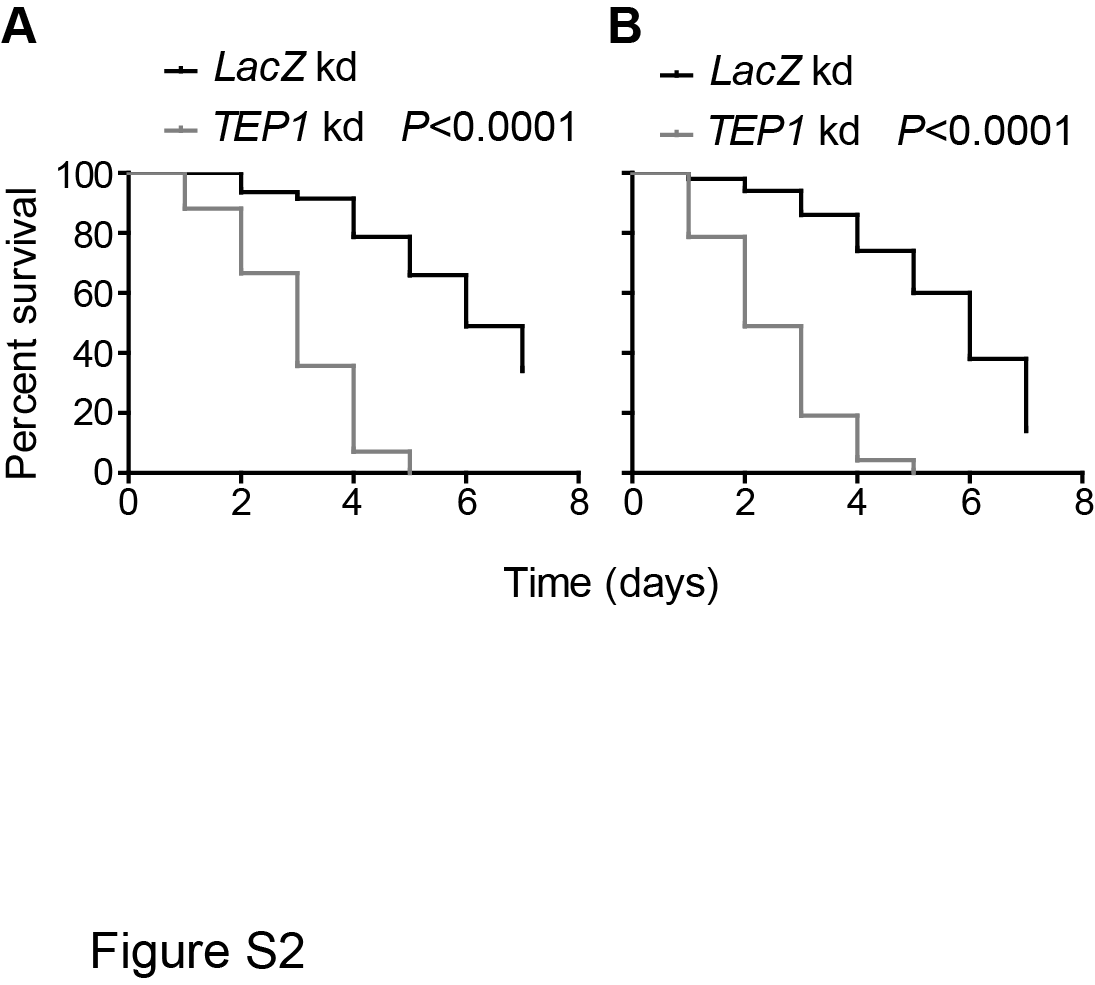

Supplement: Figure S2 — TEP1 -silenced aseptic mosquitoes are still sensitive to B. bassiana infection. LacZ and TEP1 kd female A. gambiae mosquitoes treated with antibiotics, to eliminate or at least reduce substantially their microbial flora, were challenged with B. bassiana (strain 80.2) by spraying mosquitoes with a suspension of 1×10^8^ conidia/ml. Dead mosquitoes were counted daily over the indicated period. (A) and (B) Two independent experiments performed with different batches of mosquitoes and fungal conidia. Graphs represent percent survival as calculated by the Kaplan-Meier method. Statistical significance was calculated by the log rank test. Survival curves were considered to be significantly different if P<0.05. (TIF) [file ppat.1003029.s002.tif]
